# The Prevention of Purulent Conjunctivitis in the Eyes of New-Born Infants1Read before the Buffalo Medical and Surgical Association, December 2, 1890.

**Published:** 1891-02

**Authors:** Jacob M. Falk

**Affiliations:** Buffalo, N. Y.; Attending Surgeon in charge of the East Side Eye, Ear and Throat Dispensary; 415 Pearl Street


### THE PREVENTION OF PURULENT CONJUNCTIVITIS IN THE EYES OF NEW-BORN INFANTS.[1]

By JACOB M. FALK, M. D., Buffalo, N. Y.

Attending Surgeon in charge of the East Side Eye, Ear and Throat Dispensary.

Purulent conjunctivitis of new-born infants (Syn. ophthalmia neonatorum; blennorrhea neonatorum) is a most frequent cause of blindness in infancy. The study of this subject in early life, and especially the examination of the young pupils in institutions for the blind, proves conclusively that one-third of all the cases of blindness were acquired in childhood, and lost their vision from blennorrhea neonatorum.

Dr. Reinhardt found among 2,168 persons present in twenty-two different institutions for the education of the blind, in Germany, Holland and Denmark, 658 of the inmates, nearly one-third of the number, blinded by ophthalmia neonatorum. Professor Magnus found thirty-four per cent. of similar cases in the institution at Breslau. Such facts as these show satisfactorily the great danger which threatens, not only the children of a country like Germany, but the children of every nation, where neglect and carelessness have been present at birth, and no steps had been taken to prevent the resulting dire consequences. If a tabulation of total blindness be arranged, according to the causes which operated from every direction to bring about this condition, it will be found that the conjunctival diseases played the most important part. In such a classification of 2,528 cases of total blindness, made by Professor Hugo Magnus, those of ophthalmia neonatorum represented

1. Read before the Buffalo Medical and Surgical Association, December 2, 1890.

10.876 per cent. of this number; and the other contagious forms of the conjunctiva were the cause of 240 cases of total blindness, or 9.492 per cent. Similar investigations made in our blind institutions in this State, at New York City and at Batavia, show that twenty per cent. of these cases of blindness were caused by a neglected treatment of purulent ophthalmia. The United States census of 1880 gave as the total number of blind nearly 50,000, and to this figure we can attribute at least 15,000 blind from resulting ophthalmia neonatorum. In Great Britain, in 1888, the total number of blind was 23,000, and 7,000 of these were blind from the same cause.

In the scientific sense of the term, every eye is blind which has not the capability of objectively perceiving light. The blindness can involve one eye or both eyes. For practical purposes such a person is blind whose vision is so reduced that he is prevented from following every means of livelihood where the eyes are necessary. The essential fact is his inability to maintain himself and his dependence on others for support. According to Professor Schmidt-Rimpler, all such persons are practically blind, so far as the use of their visual impressions are concerned, that can only count the figures in the nearest proximity to their eyes—numerically expressed at 1-3 m. or 13″.

All forms of purulent conjunctival inflammation occur mostly from infection. In the majority of cases of ophthalmia neonatorum the infection is caused by the vaginal secretion of the mother, remaining attached to the eye-lashes and edges of the lids of the infant, during its passage through the vagina. As the child is delivered with closed eyes, very little secretion is apt to enter into the conjunctival sac itself, but as soon as it makes efforts at repeated openings and closures the material can find entrance to the conjunctiva. An infection were possible if the passage of the head were slow, or forceps were used by displacement of the soft parts, and thereby opening the lids. Cases are known of an immediate outbreak of the disease, and in one case there was perforation of both corneæ in consequence of an infection intra-utero, caused by the manipulations of the accoucheur carrying the virulent secretion of the vagina onto the face of the child.[1]

Purulent conjunctivitis of new-born infants is not only caused by a virulent vaginal secretion, but a simple catarrhal affection

1. Undoubtedly this was a case of face presentation. King's Manual of Obstetrics says: "In the treatment of face cases avoid rupturing membranes during examinations in early stage, and beware of injuring the eyes with the finger."

accompanying pregnancy would also cause a light form of inflammation. If infection takes place at birth, the disease manifests itself on the second or third day thereafter, the difference depending on the character and the quantity of the secretion which entered the eye. If inflammation sets in later than five days, then infection, no doubt, took place after birth by reason of the dirty manipulations with unclean fingers and sponges of the nurse; but such forms are simple. Inflammation of the other eye takes place in a great many cases, and it is, no doubt, owing to a direct infection from the first eye.

In lying-in and foundling institutions the disease is often carried by the carelessness of the personnel from one nursling to the other. In this way many babies were formerly infected. In the Vienna Foundling Hospital there were from the years 1854 to 1866, 5,616 children sick with ophthalmia, and of these more than 1,400, twenty-five per cent., first obtained the disease in the institution itself. In former years, when the cause of the disease was not yet well understood, it was thought that certain unknown agents aided in the infection, as epidemics of conjunctival blennorrhea were sometimes very active in the foundling institutions; no doubt, the hands and instruments of the accoucheur were the probable sources of infection.

These unknown factors are now well-recognized elements as a cause of virulent ophthalmia neonatorum. The microscope today can demonstrate the presence of the gonnococcus-Neisser as the cause of blennorrhea, and where it is not found, no actual blennorrhea exists. The gonnococcus, or diplococcus from a thin line of separation appearing to divide it at its centre, is always present in gonorrheal secretion. These microörganisms are characterized by their generally grouping themselves in smaller or larger numbers upon the surfaces of the pus cells, or within their interior.

Purulent ophthalmia in new-born children and the gonorrheal conjunctivitis of the adult, produced by infection from gonorrheal secretion, are both closely related in their anatomical changes. Both present a high degree of inflammation of the mucous membrane, with copious purulent secretion, in both forms the micro-organisms of Neisser being present, these having been identified by Professors Haab and Sattler as identical with such as are found in the secretions of urethral and vaginal gonorrhea. From this typical form of ophthalmia there is also a simple purulent form, in which these morbific germs are entirely absent. We have thus two forms of inflammation in the eyes of the new-born infants: one having more the character of a simple catarrh, the other presenting all the phases of a virulent, purulent inflammation of the conjunctiva.

In the University Institution of Berlin the per cent. of blennorrhagic children to the total number of new-born, ranged in a period of years from 1830 to 1869, between one per cent. and eight per cent.; whereas in the Charitè, also located in Berlin, the cases of blennorrhea from 1817 to 1879 made up an average of seven to twenty-one per cent. In the foundling hospitals the average number of cases is yet larger:

| | | |
|---|---|---|
| Prague | 1865-68 | 8.6; 13 per cent. |
| Vienna | 1856-66 | 4.0; 31 per cent. |
| St. Petersburg | 1830-78 | 5.9; 10 per cent. |

But in these last cases twenty-five per cent. can be considered as infected from the other children after entering the institution. Outside of the public institutions ophthalmia neonatorum is seldom found among the educated classes, more often among the poorer people, as cleanliness in all matters is not so strictly observed among them. The condition of the child is often neglected by the midwives, and even the parents are negligent of its care; it is only the severest cases which are brought to the clinic for treatment. Hirschberg, of Berlin, with a material of 21,000 cases of miscellaneous eye diseases, had only 320 cases of ophthalmia thereunder.

Of the dangers attending neglected cases, and especially in practice among the general public, where ignorance of the consequences involved is only surpassed by the methods of treatment employed, which hasten events, failure on the part of the parents to call in a physician, or the nurse to suggest having the eyes of the infant treated, are often the cause of many of the severest forms of ophthalmia, with damage done to the cornea, and the sad results following therefrom. A tabulation of some such cases properly treated and other cases neglected, illustrates this fact quite effectively. Of 100 cases of ophthalmia neonatorum treated at the Cincinnati Hospital in a proper manner, and another series of 100 cases brought to the hospital at different stages of the condition, only fifty-eight of these were free from corneal implications; six were blind in both eyes, and five in one eye. The 100 cases treated methodically all recovered. Hirschberg, in the course of six years, treated 200 cases, among which there were already, when first coming under observation, fifty-five cases of corneal complication and six eyes totally lost (See Table No. 1).

The prophylaxis of blennorrhea neonatorum, or the prevention of purulent conjunctivitis in new-born infants, belongs to the domain of hygiene, and this most important office, if thoroughly conducted under its auspices and well performed, is most often successful in giving happy and thankful results. It was a natural consequence of the supposition that blennorrhea depends for its inception upon infection that the method pursued at the Basel Institution by Professor Bischoff, as early as 1875, was the disinfection of the vagina with carbolic acid solution, and the eyes with salicylic acid; since then many other lying-in hospitals have been using this or other methods to serve the same purpose. To do its full duty, prophylaxis must prevent, as far as possible, infection during birth as well as after birth. During the period of the mother's pregnancy, if there be a catarrh of the vagina, more especially of a purulent character, it must be attended to; where this is not done, thorough disinfection at birth must take place.

Credé, in his first publication on this subject, laid principal stress on the disinfection of the vagina, and after birth cleansed the eyes with boracic acid solution. In Austria the midwives are compelled to disinfect the vagina before birth. Of what importance in itself this act of disinfection has in preventing ophthalmia neonatorum, we are not able from statistics to prove anything, as the disinfection of the eyes takes place at the same time. Today the most attention is devoted to the disinfection of the eyes of the child. The infectious secretion generally enters the eyes after birth; distributed along the lid edges and eye-lashes it soon gets into the conjunctival folds after a few repeated movements of the lids. This can be prevented by cleanliness; even after the most active material has been deposited for a short time within the conjunctival sac, careful cleansing and removal will prevent infection.

The different methods which have been tried are: The simple washing of the eyes with pure water; with this method the number of cases have been slightly reduced by some, but water is not a trustworthy agent when used alone. The cleansing with disinfecting solutions; Credé began his first attempts with a borax solution (1 to 60), but was dissatisfied with his results; others used salicylic acid, chlorine water, but the larger number used two per cent. solutions of carbolic acid, and not stronger. Carbolic acid has a great advantage in the fact of being most constantly on hand, and the preparation ready for the physician's use.

*The Instillation of Silver Nitrate.*—After Credé met with such unsatisfactory results in the use of borax solutions, he experimented with different modes of treatment, especially testing the property of silver solutions in varying strength to combat the danger of infection, and came at last to employ the method now mostly used today, and known as Credé's method. He conducts it as follows: After ligation of the umbilical cord the child is transferred to the bath, and with clean water the eyes are cleansed of adhering secretions. Hereupon the child is laid flat, and into each eye, by means of a glass rod, one drop of a two per cent. solution of silver nitrate (ten grains to the ounce) is allowed to fall directly onto the cornea. Other further attention the eyes do not receive.

One would think that such a strong solution of silver would do damage to the eyes of an infant; but Credé, and those who follow his method, say that, in most cases, there is no reaction from the use of it. In some cases hyperemia and some secretion of the conjunctiva was present, but after three days these symptoms disappeared without treatment. In these cases the therapeutical action of the silver solution is regarded as an antiseptic agent, the solution coagulating the albuminous matter present, and entering into chemical combination with it; the effect is more than superficial. It causes an eschar among the upper layers of the conjunctival epithelium, so that if the microörganisms have already obtained entrance, the silver solution will reach and destroy them.[1]

Professor of Ophthalmology, Alfred Graefe, at Halle, was one of the first to raise his voice in favor of prophylaxis of the eyes of new-born infants, and in 1878 was successfully using a two per cent. solution of carbolic acid for treatment in his clinic; he also interested his colleague, the obstetrician, Dr. Olshausen, to use his method for antiseptic prophylaxis in his department.

Professor Graefe, although admitting the wonderful results of a two per cent. solution of silver, has grave apprehensions that such a strong concentration upon a healthy and all-succulence-devoid conjunctiva, might lead to diffuse scar tissue upon the surface of the same, or an inflammation of the cornea also take place, owing to the protracted reaction which usually ensues upon such an application.

---

1. In an article on the Relative Germicidal Value of the So-called Antiseptics, by John E. Weeks, M. D., in the *Medical Record* of August 3, 1889, he says: "Nitrate of Silver: In this salt we have an excellent germicide, the value of which does not appear to be recognized by the profession. Aside from the work by Ratincoff and Miquel, who determined that in the proportion of 1 to 10,000 it arrested putrefactive processes, little has been done with the nitrate of silver in an experimental way. We may assign this salt to a position near the head of the list of germicidal remedies." This explains its great efficacy in the treatment of eczema, purulent affections of the eye, otorrhea, etc.

Every new method of treatment is put to the test by the results obtained. Statistics gathered of the absolute number of observed cases of ophthalmia neonatorum and their per cent., under methods pursued before prophylaxis was employed, and after the use of different antiseptic agents, as employed by different authorities, show varying important differences. These data are of interest in exhibiting results obtained by the use of water, a two per cent. solution of carbolic acid, and by the use of a two per cent. solution of silver nitrate. (See table before prophylaxis and after the use of prophylaxis.) From such facts it will be readily seen that the disinfection of the eyes immediately after birth, and done with care, is a most efficient means to protect the child from the dread effects of blennorrhea. This should be done in the case of every infant.

A very interesting experiment of Königstein, (an eye specialist of Vienna,) who, arranging his cases into those of a simple catarrh and of blennorrhea, watched the history of 1,092 infants, which received no treatment; then noted a second series of 1,541 infants, where he used a one per cent. solution of carbolic acid; and in a third series of 1,250 infants, where he used a two per cent. solution of the nitrate of silver. All these observations and experiments took place at one clinic, conducted by the same set of attendants, and the results tabulated by the same individual, so that these observations represent facts, and have scientific worth. Königstein's results as tabulated are:

| | New-born. | Blennorrhea, Per cent. | Catarrh, Per cent. | Total. |
|---|---|---|---|---|
| Without treatment | 1,092 | 4.76 | 14.50 | 19.26 |
| One per cent. carbolic acid | 1,541 | 1.42 | 6.00 | 7.42 |
| Two per cent. silver | 1,250 | 0.72 | 4.72 | 5.44 |

In Germany most cases of delivery are conducted by educated midwives; not even among the best families does the doctor appear until absolute necessity requires his management of a difficult case, much less does he appear among the poor. In Sweden, where there is a great deficiency of doctors, the midwives are permitted to use the forceps. To these midwives in Germany and Austria is intrusted the task of following out the strict, printed directions of antiseptic prophylaxis issued by the Government, and the good results achieved thereby are well worth all the necessary extra preparation and care essential to success.

The prophylaxis is more important in general practice, owing to the total number of births among the public being far larger than the smaller number born in institutions. Cases of blennorrhea or ophthalmia occurring among the people are always more apt to lead to bad results from neglected and improper treatment.

Upon you, gentlemen, general practitioners, whose work lies among the public, meeting every phase of life as you are called upon to attend at the birth of a child, — upon you devolves the important duty of careful watching and prevention of this dread disease at the births which come under your personal care.

TABLE No. 1.

| *Institution.* | *No. of cases of oph.neon.* | *Corneal Opacity.* | *Blindness one eye.* | *Blindness both eyes.* | *Per cent. of Injured Eyes.* |
|---|---|---|---|---|---|
| Charité Berlin Lying-in Hospital | 213 | 2 | 2 | ? | 1.9 |
| Munich Lying-in Hospital | 123 | 1 | 2 | 1 | 2.4 |
| Dresden Lying-in Hospital | 1,378 | 38 | 15 | 4 | 3.8 |
| Stuttgart Lying-in Hospital | 538 | 13 | 12 | 1 | 4.6 |
| Cincinnati General Hospital (first series) | 100 | 0 | 0 | 0 | 0 |
| Cincinnati General Hospital (second series) | 100 | 42 | 5 | 6 | 53.0 |
| Vienna Foundling Hospital | 1,347 | 112 | 171 | 42 | 21.0 |
| Prague Foundling Hospital | 300 | 105 | 32 | ? | 45.7 |
| *Private Clinics.* | | | | | |
| Hormer | 108 | 43 | 0 | 0 | 39.8 |
| Hirschberg | 200 | 55 | ... | 6 | 27.5 |
| Emrys Jones | 420 | 72 | ... | 16 | 17.1 |

TABLE No. 2.

| *Author.* | *Before Prophylaxis.* | | | *After Prophylaxis.* | | | |
|---|---|---|---|---|---|---|---|
| | *Total No. of Cases.* | *Cases of Blen.* | *In per cent.* | *Prophylactic Method.* | *Total No. of Infants* | *Cases of Blen.* | *In per cent.* |
| Abegg | ...... | ...... | ...... | Pure Water | ...... | ...... | 3 |
| Bischoff | ...... | ...... | 5.6 | Salicylic Acid | ...... | ...... | 2.6 |
| Olshausen | 550 | 69 | 12.5 | Two per cent. Carbolic Acid Solu. *after* ligation of umbilical cord. | 137 | 12 | 8.8 |
| Olshausen | ...... | ...... | ... .. | Two per cent. Carbolic Acid Solu. *before* ligation | 166 | 6 | 3.6 |
| Krukenberg | 1,266 | 92 | 7.3 | Two per cent. C. A. Solu. before ligation | 82 | 11 | 13.4 |
| Königstein | 1,092 | 51 | 4.8 | One per cent. C. A. Solu. before ligation | 1,541 | 21 | 1.4 |
| Credé | 2,897 | 314 | 10.8 | Two per cent. Argentum Nitricum 10 gr. to oz | 1,160 | 2 | 0.2 |
| Königstein | ...... | ...... | ...... | " " " | 1,250 | 9 | 0,7 |
| Krukenberg | ...... | ... .. | ...... | " " " | 703 | 1 | 0.14 |
| Felsenreich | 1,887 | 82 | 4.3 | " " 1st series. | 3,000 | 58 | 1.9 |
| Felsenreich | ...... | ...... | ...... | " " 2d " | 2,100 | 21 | 1.0 |
| Bayer | 1,106 | 136 | 12,3 | " " " | 361 | 0 | 0 |

TABLE No. 3.

| *Königstein's Method and Results.* | *Total No. of Infants.* | *Blen. in per cent.* | *Catarrh in per cent.* | *Total per cent.* |
|---|---|---|---|---|
| Without treatment | 1,092 | 4.76 | 14.5 | 19.26 |
| One per cent. Carbolic Acid Solution | 1,541 | 1.42 | 6 | 7.42 |
| Two per cent. Argentum Nitricum 10 gr. to 1 oz | 1,250 | 0.72 | 4.72 | 5.44 |

415 PEARL STREET.

